# Exploring the Sociodemographic Factors and Consequences related to Alcohol Consumption among Older Indigenous Community of a District in Nepal: A Qualitative Study

**DOI:** 10.31729/jnma.8495

**Published:** 2024-03-31

**Authors:** Salina Begam, Shishir Paudel, Anisha Chalise, Gulam Moiz Khan, Lirisha Tuladhar, Santosh Khadka

**Affiliations:** 1 Department of Public Health, CiST College, Pokhara University, New Baneshwor, Kathmandu, Nepal; 2 Center for Research on Environment Health ana Population Activities (CREHPA), Kusunti, Lalitpur, Nepal; 3 Faculty of Health Sciences, School of Health and Allied Sciences, Pokhara University, Lekhnath, Kaski, Nepal

**Keywords:** *alcohol consumption*, *alcohol drinking habits*, *alcoholism*, *cross sectional study*, *qualitative research*

## Abstract

**Introduction:**

Alcoholism is a major global public health concern associated with numerous health conditions. Alcohol use has been a cultural part of several ethnic groups in Nepal. This study aimed to explore the qualitative dimension of alcohol use, its promoting factors, and consequences in Nepalese communities.

**Methods:**

Qualitative study was conducted among 20 older adults belonging to the Magar community of Mathagadhi Rural Municipality, Lumbini Province, Nepal after acquiring ethical approval from Institutional Review Committee of CiST College (Reference number: 179/078/079). The data were analyzed using inductive thematic analysis, and themes were identified based on participants' responses to explore promoting factors for alcohol consumption along with its consequences.

**Results:**

Traditional beliefs, cultural practices, and socioeconomic factors were the major contributors to alcohol misuse. Increased alcohol consumption during old age was perceived to be associated with body pain, tension, painful life events, and loneliness.

**Conclusions:**

A conflicting perception was observed, where some of the participants expressed the need to promote alcohol use as a part of their culture while some shared the view that the use of alcohol as a cultural practice should be limited. This study highlights the need for culturally appropriate interventions to address alcohol misuse among indigenous communities. Interventions should focus on addressing traditional beliefs and cultural practices that normalize alcohol consumption and the social and economic problems associated with alcohol misuse.

## INTRODUCTION

Alcoholism is a global public health concern, three million deaths worldwide in 2022, constituting 5.3% of total global deaths.^1^ In Nepal, alcohol ranks as the eleventh leading risk factor for Disability Adjusted Life Years.^2^ Magar ethnicity is one among 59 indigenous communities of Nepal comprising 6.9% of the national population, is culturally accepting of alcohol.^3,4^

Despite its adverse health effects, alcohol use is normalized, especially among males aged 45-65 in certain ethnic groups, Magar community being one of them.^5,6^ Older adults, more susceptible to alcohol's negative health impacts, face increased vulnerability.^7,8^ Existing studies in Nepal primarily focus on quantitative assessments, neglecting the qualitative dimension of alcohol use, its consequences, and enforcing factors among Nepalese ethnic groups.^6,9^

Thus, this study aimed to meet this research gap and explore the factors contributing to alcohol misuse and its consequences among the older adults of the Magar community.

## METHODS

### Study Design and Setting

A qualitative study design was adopted to explore the factors contributing to alcohol misuse and its consequences among the indigenous ethnic group of Nepal from the month of January to March 2023. The study was conducted in Mathagadhi Rural Municipality of Palpa district which is located in Lumbini Province of Nepal. The study site was chosen purposively due to the availability of the native Magar community.

### Sampling Techniques

The older adults above the age of 60 years, belonging to the native Magar communities of the Palpa district were considered as eligible participants for the study. There is no exclusion made in terms of any sociodemographic or other characteristics. A total of twenty older adults were interviewed for the study where they were sampled considering diverse participants based on gender, education status, and sub-ethnicity of Magar communities. The number of interviewees was determined by data saturation; subsequent interviews did not yield any new information.

### Data Collection

The consolidatedcriteria for reporting qualitative studies were followed (S1 : COREQ checklist). The interview guideline was developed by the first author and was reviewed and approved by the second, fourth, and last authors. It was piloted among three older adults belonging to the Magar ethnic group in Mathagadhi Rural Municipality, Palpa who were not included in the original sampling frame. The first author amended the guideline considering the feedback collected during the piloting.

All twenty in-depth interviews were taken in-person with the interviewees at their residence by the first author. The interviewer briefed the objective of the study and developed some rapport with the participants to make them at ease. All the participants agreed to the face-to-face interview and provided their complete responses to all the questions. All the interview sessions were of almost an hour. The interviews were recorded after acquiring informed consent from the participants and the field notes were also taken during the interviews by the fourth author. Participants were expressive and comfortable communicating in the Nepali language, so all the interviews were conducted in the Nepali language. The answers which were not clear were asked again with the participants. The important and interesting observations were noted in a notebook by the fourth author. The recorded audio was transferred and kept safely in a hard drive and was deleted from the recording device. The recordings were pseudonymized as R1, R2, R3, and so on to ensure confidentiality.

### Qualitative Data Analysis

All in-depth interview audios were transcribed verbatim on Google Documents by two authors (SB and GMK). Each of the files was saved with appropriate pseudonymized file names. The senior authors (SP, AC and SK) cross-checked the transcripts for accuracy and language translation consistency from Nepali to English. Prolonged engagement of all the authors in every phase of the study was ensured and peer debriefing of translations of verbatim was done. The transcripts were not sent to the participants for their review. To fully comprehend the subject matter and to have a thorough understanding of the participants' feelings and experiences, the interview notes and transcripts were read and analyzed numerous times. NVivo software was used to organize and analyze the translated text data of the interview into codes.

The inductive thematic analysis approach was used where the author (SB) independently generated the initial codes based on the essential ideas shared by each participant in their interviews. The concepts were clustered into themes based on their similarities to develop a comprehensive description ofthepartici pant's expressions. The other authors (SP and SK) reviewed the codes and collaboratively defined and named the themes. In case of disagreements in the codes and themes generated by SB and reviewed by SP and SK, meetings were held and the next author (AC) worked as the mediator to finalize the codes and themes where there were some disagreements. All authors collaboratively reviewed and finalized the themes.

A general codebook was developed to categorize the texts more easily and maintain the consistency of the coding. The codes generated were input into the NVivo software and coded from the texts about the context of data. Caution was maintained to distinguish between important and less important information based on the aim of the study and the coding framework. The open codes generated were compared with each other based on the similarities and their differences and taken further for the process of axial coding where the codes were further scrutinized and categorized into much more concise categories by the second, fourth, and corresponding authors. After this, the codes sharing common features were grouped to develop the sub-themes,anda similar sub-theme wasarrangedtodevelop a generated thematic map. The author (SB) performed the thematic analysis which was revised with the suggestions of the remaining authors. The authors (SB, GMK, LT) are health science undergraduates with prior engagement in health research whereas the authors (SP, AC and SK) are the public health postgraduates with years of experience in both quantitative and qualitative research methodology.

### Ethical Consideration

The ethical approval for this study was obtained from the Institutional Review Committee of CiST College (Registration number: 179/078/079). Informed verbal consent was obtained from all the participants before they participated in the interview. The interview session was recorded after obtaining permission from the participants and all the information of the participants revealing their identity was kept confidential using participant code.

## RESULTS

A total of 20 participants were interviewed, of which there were 11 (55%) male and 9 (45%) female participants. Nearly half of the participants 8 (40%) belong to the Thapa sub-ethnic group (Table 1).

**Table 1:** Demographic characteristics of the participants (n=20).

Characteristics	n (%)
**Marital Status**	
Married	10 (50)
Widow	10 (50)
**Occupation**	
Farmer	13 (65)
Indian Army	4(20)
Labor	3(15)
**Income**	
Dependent	14 (70)
Independent	6(30)
**Sub-ethnicity**	
Ale	5(25)
Gharti	3 (15)
Rana	4(20)
Thapa	8(40)

### Qualitative Findings

**The situation of alcohol consumption**

Out of the total of 20 participants, 11 (55%) older adults reported that they still consume alcohol daily while 9 (45%) of the older adults reported to have stopped consuming alcohol due to some health issues. The participants reported that they initiated the drinking habit during their childhood as part of their rituals during festivals and occasions. They shared that there is a belief among the community members that any person belonging to the Magar community must drink a little amount of homemade alcohol as a ritual which can lead to continuous intake of alcohol further guiding towards alcohol habituation.

*"We have a ritual of consuming homemade alcohol on all occasions. After marriage, there is a practice of taking curd, meat and alcohol as a ritual. Nowadays, some people ask to bring beer as well. I started drinking alcohol when I was a little child as my parents used to give jaad whenever they used to drink it. So, I also fed my grandchildren in the same way from their childhood. But they don't drink now. " - a 65-70 years old male participant*

A total of 12 (60%) participants also revealed that they prefer homemade alcohol in comparison to other alcoholic beverages because it is easily available at home and also they like the taste of homemade alcohol. The participants also shared that alcohol is a part of their daily routine and they need to have a drink in the evening before going to sleep.

*"...I drink alcohol on a daily basis. I also drinkjaad as a source of food. I usually prefer home-made alcohol over market one as I like the taste of home-made alcohol and we don't need money to buy it." - a 65 years old male participant*

Out of eight participants who stopped consuming alcohol, 6 (75%) shared that they experience severe health effects such as high blood pressure, liver disease, heart disease, and digestive problems which lead them to quit the habit.

*"...alcohol has severe effects on health. Whenever I used to drink, it increased my blood pressure. Recently, one of my cousins died due to alcohol. He drank alcohol on the hot sunny day during the afternoon and as alcohol produces more heat in the body, he got unconscious suddenly and died, he didn't have any health problems." - a 65-70 years old male participant*

**Social and cultural aspects of alcohol**

Homemade alcohol has a cultural value in Magar communities as it is used in several rituals and almost at every festival and occasion including the funeral ceremony. The older adults reported that alcohol must be provided to the guests as a sign of hospitality as well.

*"Homemade alcohol is a must in every occasion of Magar household. Without alcohol, every festivals and occasions feel incomplete. We must also provide our guests with alcohol while they visit us as alcohol is taken as a sign of hospitality and good social relationships." - a 70-75 years old female participant*

The participants also highlighted the fact that with changing lifestyles, the community people have started to dislike this culture of alcohol use as they acknowledge the fact that alcohol consumption has hampered people's daily activities and their health. This has led to a significant fall in alcohol consumption as a part of cultural and traditional practices. Thus, conflicting perceptions were placed by the participants where nine of the participants expressed that they need to promote alcohol use as it is a part of their culture which needs to be saved while 7 (35%) participants shared the thought that some of the cultures are better to be modified and the alcohol misuse should be limited.

*"...homemade alcohol is a must on every occasion. We need to provide it to our guests and it is compulsory to drink during the festivals and occasions as it is our culture from very long years ago so this should not be stopped." - a 60-65 years old male participant*

*"...alcohol is compulsory during our festivals and occasions. But we don't follow this culture nowadays. We shouldn't follow such a culture. I always wanted to stop this so I initiated and didn't give alcohol at my father's funeral ceremony. As a result, all the villagers got angry at me for that. We should follow only the right culture and stop following bad culture,* "-a 65-70 years old male participant

**Perception towards the ill effect of alcohol consumption**

Out of 20 participants, 16 (80%) felt that although there is a ritual of drinking alcohol on every occasion, alcohol is bad for their personal health and social relationships. They shared that alcohol consumption causes different health-related effects like raised blood pressure, liver disease, heart disease, and digestive problems and also can lead to loneliness and depression.

*I started drinking with my family members when I was about 10 years old and stopped drinking at the age of 60 years as alcohol lead to high blood pressure and heart problem due to which I was about to die. The doctor told that if I don't stop drinking then I won't be able to survive. Then, I realized that alcohol has bad effects on health and stopped drinking with strong determination. "-* a 65-70 years old male participant

*I get irritated and angry if anyone talks to me when I am drunk so I like sitting alone while drinking and go to sleep."* - a 60-65 years old female participant

All of the participants shared that alcohol consumption has some socioeconomic effects as it has led to quarrels and also has led to economic loss in many households.

*"Whenever I used to drink, I usually got aggressive so easily and quarreled with my wife. Sometimes, while drinking with friends, we used to remember the old incidents of being hurt by each other and start blaming others. Eventually, we start to fight as we lose our consciousness. So, alcohol can create disturbances in the society. "-* a 65-70 years old male participant

*"Alcohol can cause economic loss and reduces the prestige in the society as well. After having a habit of drinking, people start to spend money to buy alcohol which often creates conflicts among the familymembers for spending more money for alcohol and this causes disturbance in the society and reduces the prestige."-* a 75-80 years old male participant

**Reinforcing factors for alcohol consumption**

Different physical, mental, and social factors were found as contributing factors to drinking alcohol among older adults of the Magar community. In regards to physical factors, it was expressed that they drink alcohol when they feel hungry, or thirsty and to reduce body pain and tiredness after work.

*"When I was 32 years old, I got into an accident while fighting due to which I had to go through severe pain so as not to feel that severe pain I drank alcohol. At that time, I drank a lot of alcohol. After that, I used to drink one bottle of alcohol daily and it became my habit."*-a 65-70 years old, male participant

Likewise, the female elderly also shared that they started drinking after they gave birth to their babies as it benefits the health of the mother and child by providing the required nutrition and also helps in milk production during the lactation period.

*"When I gave birth to my first child, my mother-in-law gave me to drink jaad. She used to say that jaad helps in milk production, makes the body warm, and keeps the body pain away. After that, I started drinking regularly."* - a 70-75 years old female participant

Among the elderly, 4 (20%) also suggest that alcohol consumption also contributes to their mental health and well-being as alcohol helps them forget all their worries and stress, making them less tense, reducing their suffering, and providing them with pleasant sleep.

*"I lost my husband about one and half years ago, then I started drinking in huge amounts to forget about the tensions, feel relaxed and get a pleasant sleep."-* a 6065 years old female participant

*"After my husband's death, I was so worried that to forget all the sufferings and pain, I started drinking alcohol and now I drink regularly."* - a 70-75 years old female participant

The participants also reported that alcohol helps them bond with their family and friends as it plays an important role in social gatherings. It creates a sense of belonging among them making it the most preferable drink when meeting friends.

*"I often drink with my peers while going to do the agricultural work in fields as it gives a pleasant feeling to drink with friends and helps in maintaining social relationships, "-a* 65-70 years old female participant

**Strategies adopted to control alcohol consumption**

Among 20 participants, 9 (45%) of the participants reported to have controlled their drinking habits by adopting different strategies like increasing the volume of water and tea consumption, reducing the amount of alcohol day by day, and also being mentally strong and determined not to follow the cultural practice of alcohol use.

*"It was quite difficult for me to stop drinking. Whenever I felt like drinking, I used to drink water and used to keep myself busy by listening to the radio and roaming around the fields. "-* a 65-70 years old male participant

*"Alcohol causes severe health problems. I have seen many people who drink alcohol being infected with severe diseases like liver disease, heart problems, and high blood pressure. I myself had high blood pressure and liver disease due to alcohol. After that, we don't follow this culture anymore in our family."-* an 85-90 years old male participant

**Support system: a key to cope with alcoholism**

The elderly people were asked about the roles of external support from the family and local government in the management of alcohol consumption.The participants acknowledge that the easy availability of alcohol in the family is one of the reasons which promoted alcohol misuse. It was highlighted by almost all the participants that if the family members don't prepare alcohol at home then its use is limited. The participants also shared that they have asked their relatives not to bring alcohol while visiting them, which has helped them control their consumption patterns.

*"Alcohol, being available at home, used to make me feel like drinking in a little amount during the evening. My family members don't like me to drink alcohol. So, my daughter-in-law doesn't make alcohol at home anymore."* - a 75-80 years old female participant

The participants also shared that the local government can bring policies, programs, and aware people to reduce the use of alcohol among children and adolescents to make them less dependent on alcohol.

*"Local government can play an important role to reduce the use of alcohol. The local government should make policies, and programs to aware people of the harmful effects of alcohol so that people will reduce drinking alcohol."-* a 65-70 years old female participant

## DISCUSSION

In this study, we aimed to explore the major contributors for alcohol consumption among Older adults of Magar community. Among the twenty participants, eleven reported daily alcohol consumption, while nine had ceased drinking due to health issues. Participants highlighted the cultural significance of homemade alcohol in rituals and social gatherings, emphasizing its role as a sign of hospitality and tradition. Conflicting perceptions regarding alcohol use were evident, with some advocating for its preservation as a cultural practice, while others emphasized the need to modify or limit alcohol misuse. The study suggests that the majority of older adults in the indigenous community still consume alcohol regularly. A past study from Nepal also suggested that older adults have higher odds of alcohol consumption than the youth with higher alcohol use in disadvantaged ethnic communities.^6^ It was observed that alcohol is preferred as a part of the culture on most of the occasions such as festivals and funeral ceremonies among Magar community. Similar observation was shared by the previous study aimed to study the Ritual and Behavior of Magar community where the community people reported the use of homemade alcohol during the worship of clan deity.^3^ Supporting this phenomenon, a qualitative metasynthesis based on studies among Indigenous peoples of Colombia also suggested that alcoholism has been a social part of indigenous communities in Colombia.^10^ Similar observation was also shared by a quantitative study from a national epidemiologic of alcohol and related conditions among older U.S. adults where alcohol was the most commonly used substance over their lifetime and is still the substance of choice of the older adults.^11^

It was also observed that the indigenous community mostly preferred to drink homemade alcohol. Past studies from Nepal focusing on the alcohol consumption pattern among Nepalese women have also noted that the proportion of women consuming homemade alcohol is almost double than that of those consuming an industrial product in Nepal.^12^ This suggests that a significant proportion of the Nepalese population prefers homemade alcohol, generally termed as 'Jaad' and 'Raksi'. The most common reason for their preference over homemade alcohol can be its easy access as it is prepared by themselves at home, is cheaper, and is in abundance. The connection between the cost and availability of alcohol and its consumption has also been noted by a systematic review, where it was concluded that cost and availability could heavily influence drinking behavior in older people.^13^

The older adults of the indigenous community were found to perceive alcohol consumption as an important part of their culture making alcohol a highly accepted part of the community. This acculturation of alcohol among indigenous communities has also been noted by another study from Nepal on liquor and culture in Nepalese society revealing how culture has shaped the alcohol use among indigenous people.^14^ Alcohol is often found in every religious occasion and celebration among the Magar community. For instance, there is a ritual of "Duran" in which the newly married couples are expected to go to the bride's home with a bottle of wine and goat leg.^15^ Alcohol has been reported as a part of culture in other countries as well such as India^16^ and Africa.^17^ A systematic review suggested that alcohol use has been linked with social life and enhancing social engagement in studies throughout the world.^13^ A qualitative meta-synthesis also suggested that alcohol use has been normalized because it is considered part of everyday life in many societies.^10^ This has also been reported by the participants in our study.

During the interviews, a conflicting perception was observed among older adults regarding alcohol use as a culture where some of them expressed the need to promote alcohol use as a part of their culture that needs to be preserved, while others shared an opinion that some of the cultures are better to be modified. There was a strong opinion from the communities that alcohol misuse should be limited as alcohol has caused several health effects and social disturbances among the indigenous group. Evidence suggests that older people often have their own views about the social acceptability of alcohol use and disapprove the issue of excessive drinking in older people.^13^ Similar cultural changes have also been observed in other nations, where the indigenous communities are becoming more aware and hesitant about their cultural practice of normalizing alcohol use.^10^

The study suggests that alcohol consumption among the indigenous community was mostly initiated with its ritual values through the family environment and peer pressure. Likewise, a qualitative study on the indigenous tribal men in Wayanad reported that alcohol use was often initiated through parental and peer routes.^18^ It has been noted that the habit of alcohol consumption tends to be initiated by their partners, family members, and peers where this habit can become a routine of their life.^13^ Similar, habits were also reported by the participants in our interviews.

It was observed that body pain, tension, painful life events, and loneliness during old age are connected with an increasing amount of alcohol consumption among older adults in the indigenous community. This phenomenon is in line with an observation made by a systematic review notifying several studies highlighting alcohol use as a coping strategy for negative life events such as loneliness, loss of family or friends, loss of physical health and mobility, aches, and mental distress.^13^ The participants in our study revealed that consuming alcohol in little amounts provides them ease from body pain, and tiredness and is beneficial to health and this is one of the reasons that older adults prefer to drink alcohol. A national survey in Japan noted that older adults having low physical activity, unhealthy eating habits, and divorced relationships are more likely to consume higher amounts of alcohol.^19^ Also, a prospective, longitudinal study on high-risk alcohol consumption and drinking problems among older adults shared that older adults rely on alcohol use for tension reduction.^20^

The older adults in good health were found to be consuming alcohol on regular bases while those with certain health conditions linked to alcoholism were seen to be hesitant about its consumption. This phenomenon is relatable to the previous study from the United Kingdom where adults in later life were found to have continued heavy drinking as a normal behavior among those with relatively good health while less drinking behavior among those with impaired health.^21^ The participants in our study suggested that alcohol consumption has caused different health effects like high blood pressure, heart disease, liver disease, and digestive problems, and also led to quarrels, and social disturbances. Similarly, a systematic review on substance abuse suggested an increase in alcohol consumption has many health consequences such as falls, injuries,functional impairment, mental impairment, and mortality as well.^22^ In context of Nepal, multiple studies has highlighted liver cirrhosis to be one the major complication of alcohol use, which has significantly led to economic, social and mental burden to the consumer and their families.^23^'^25^

However, in our study, along with liver disease the participants primarily highlighted cardiovascular diseases (CVDs), such as hypertension and heart disease as well as mental distress as the perceived complication of alcohol use. This discrepancy might be due to various factors such as cultural beliefs, differences in alcohol consumption patterns, and/or participant poor awareness in regards to liver disease, which we failed to assess in this qualitative survey. Future research in the Magar community should explore liver cirrhosis prevalence and factors to better understand alcohol's health effects.

It was observed that even though some of the adults were eager to quit alcohol being aware of its ill effect, they were still unable to completely leave it behind. The older adults were found adopting different strategies to stop drinking alcohol like drinking tea, and water instead of alcohol, being mentally determined, and not following the culture of drinking during occasions and festivals to reduce the health effects of alcohol. Similarly, a previous study on the use and misuse of alcohol among older women shared that a brief intervention providing 5 to 15 minutes of informative sessions and advice about the risks of alcohol use and ways of reducing alcohol use can play a vital role.^26^ There is a huge need to develop preventive approaches among the indigenous group targeting older adults to limit the use of alcohol and prevent its consequences. The family and local government can act as a support system to cope with alcoholism by reducing the use of alcohol as a ritual and conducting awareness among indigenous groups about its harmful health effects respectively. The conflicting perceptions observed among participants regarding alcohol use have important implications for interventions aimed at promoting healthier alcohol consumption habits in the Magar community. Interventions need to take into account the cultural significance of alcohol and the role it plays in social gatherings and rituals. Any intervention that seeks to reduce alcohol consumption must be culturally sensitive and respectful of the community's traditions. It is also important to address the social and economic factors that contribute to alcohol consumption, such as the need for social bonding and the perception of alcohol as a coping mechanism for stress and grief. By addressing these conflicting perceptions and understanding the cultural context of alcohol use, interventions can be more effective in promoting healthier behaviors among older adults in the Magar community.

Though this is one of the few studies assessing the qualitative traits promoting alcohol consumption among old adults of an indigenous ethnic group of Nepal, this study is not free from its limitations. This study was executed during the period of the COVID-19 pandemic as a result the researcher failed to cover a larger geographic area and limited the study only to a specific indigenous group of the Palpa district. Nepal is a multiethnic country with diverse indigenous groups, and alcohol use has been historically and culturally accepted in many of these ethnic groups which were not explored in this study. However, attempts have been made to make the findings generalizable by covering diverse participants based on gender, education status, and sub-ethnicity of Magar communities.

## CONCLUSIONS

Alcohol was perceived as an important part of the culture among the indigenous community. Traditional beliefs, cultural practices, and socioeconomic factors were found to be the major contributors to alcohol misuse among the Magar communities in Nepal. Alcohol consumption was mostly initiated through the family environment and peer pressure. Physical pain, mental distress and painful life events, and loneliness during old age were linked with an increased amount of alcohol consumption. Though it is considered to be part of their culture, some of the community members are willing to limit their cultural practice of alcohol use. Different strategies were adopted by older people to stop alcohol consumption and limit alcohol use in their family but they were not very effective. The conflicting perceptions regarding alcohol use highlight the need for culturally sensitive interventions addressing the social, economic, and cultural factors influencing alcohol consumption.
